# Nonspecific interstitial pneumonia: clinical associations and outcomes

**DOI:** 10.1186/1471-2466-14-175

**Published:** 2014-11-07

**Authors:** WenBin Xu, Yi Xiao, HongRui Liu, MingWei Qin, WenJie Zheng, JuHong Shi

**Affiliations:** Division of Pulmonary Medicine, Peking Union Medical College Hosptial, Chinese Academy of Medical Sciences & Peking Union Medical College, 100730 Beijing, China; Division of Pathology, Peking Union Medical College Hosptial, Chinese Academy of Medical Sciences & Peking Union Medical College, Beijing, China; Division of Radiology, Peking Union Medical College Hosptial, Chinese Academy of Medical Sciences & Peking Union Medical College, Beijing, China; Department of Rheumotology, Peking Union Medical College Hosptial, Chinese Academy of Medical Sciences & Peking Union Medical College, Beijing, China

**Keywords:** Non-specific interstitial pneumonitis, Connective tissue disease, Prognosis

## Abstract

**Background:**

Studies have shown that nonspecific interstitial pneumonitis (NSIP), even when initially diagnosed as an idiopathic form of the disease, might be associated with an autoimmune background that later reveals itself as an organ-specific or a systemic autoimmune disease.

**Methods:**

NSIP patients were divided into three groups. The NSIP patients who met the criteria for having a systemic autoimmune disease (SAD) were defined as the systemic autoimmune disease-associated NSIP (SAD-NSIP) group. The NSIP patients who did not meet the criteria for a systemic autoimmune disease were defined as an antibody-positive group (i-NSIP-Ab + group) if their sera were positive for autoantibodies. The NSIP patients with negative serologic tests for auto-antibodies were defined as the antibody-negative group (i-NSIP-Ab- group). The clinical characteristics were analyzed and compared among the three groups.

**Results:**

Ninety-seven NSIP patients were included. The mean age of the study population was 48 ± 11 years. The mean follow-up time was 54 ± 34 months. At the time of the surgical lung biopsies, 23/97 (23.7%) of the patients were classified as SAD-NSIP; 30/97 (30.9%) were in the i-NSIP-Ab + group; and 44/97 (45.4%) were in the i-NSIP-Ab- group. At the end of the follow-up period, three cases were diagnosed with polymyositis (one case from the i-NSIP-Ab + group, two cases from the i-NSIP-Ab- group), one with scleroderma (from the i-NSIP-Ab + group, scl-70 positive and skin biopsy) and another one with microscopic polyarteritis (from the i-NSIP-AB-group, p-ANCA and MPO positive, renal biopsy). Three cases in the i-NSIP-Ab- group were later found to be positive for autoantibodies. Due to these changes in classification, at the end of the follow-up period, the SAD-NSIP group consisted of 28/97 patients (28.9%), the i-NSIP-Ab + group of 31/97 (32.0%) and the i-NSIP-Ab- group of 38/97(39.1%). There were no significant differences in clinical manifestations, radiographic findings or pulmonary function tests among the three groups at the time of surgical lung biopsy or after reclassification after the follow-up period. SAD was an independent risk factor for the survival of the patients with NSIP after follow-up.

**Conclusion:**

Follow-up is recommended because idiopathic NSIP may be the first manifestation of a systemic autoimmune disease.

## Background

The histopathologic pattern of nonspecific interstitial pneumonitis (NSIP) has been found in a wide variety of clinical contexts, including chronic hypersensitivity pneumonitis, drug-related interstitial pneumonia, and connective tissue disease (CTD) [1–5]. NSIP has been identified as one of the most common pathologic patterns in patients with CTD [6–11]. Recent studies have shown that NSIP, even when initially diagnosed as an idiopathic form of NSIP, might be associated with an autoimmune background that later reveals itself as an organ-specific or a systemic autoimmune disease [12–14]. In a cohort study of 27 idiopathic NSIP patients, more than 50% of the cases developed an autoimmune disease after a mean follow-up of 22 months [13]. Furthermore, an Asian study revealed that CTD developed in 10% of idiopathic NSIP patients during the follow-up period [14]. It has been suggested that Multidisciplinary Discussion and follow-up are especially important to establish the diagnosis of idiopathic NSIP [15, 16].

Compared with idiopathic interstitial lung disease (ILD), patients with CTD-associated ILD (CTD-ILD) had a better prognosis [17–21]. For patients with rheumatoid arthritis-associated usual interstitial pneumonia (UIP), the survival time was longer than that for patients with UIP without rheumatoid arthritis [18]. According to these studies, it might be deduced that CTD-NSIP had a better outcome than idiopathic NSIP (i-NSIP). However, the current literature is controversial. Additional studies showed that CTD did not affect the survival of patients with pathologically confirmed NSIP [22, 23]. Interestingly, the classification of undifferentiated CTD-NSIP conferred a minor prognostic advantage [23].

Given that CTD-NSIP is similar to i-NSIP on clinical and radiologic features [24–26], and if there are no differences in the disease outcomes for the two classifications, the question arises as to whether there is a value to differentiating between CTD-NSIP and i-NSIP. To answer this question, we reviewed the clinical, radiologic and physiologic findings in NSIP patients with systemic autoimmune disease and i-NSIP at the Peking Union Medical College Hospital. We wanted to evaluate whether there was a difference in prognosis for i-NSIP patients compared with those with systemic autoimmune disease in this Chinese cohort.

## Methods

### Study subjects and diagnostic criteria

Between December 2002 and December 2011, 354 patients underwent surgical lung biopsies at the Peking Union Medical College Hospital, the biggest referral center in China. The ending date for the follow-up period was December 2012. The 354 cases in this study were clinical suspicion for diffuse infiltrates. NSIP was diagnosed in 101 cases; 4 cases of drug-induced NSIP were excluded. The remaining 97 cases were enrolled in this study. Their clinical features, radiological images and pathological findings were reviewed and analyzed. NSIP was diagnosed according to the American Thoracic Society (ATS)/European Respiratory Society consensus classification criteria [1, 15]. Informed consent for using the medical records was obtained from every patient and/or their guardian when the patient was admitted to the hospital. This study was approved by Peking Union Medical College Hospital Institutional Review Board (reference number for ethics approval: 2013-9-322).

Patients with a history of drug toxicity, airborne antigen or environmental exposures were excluded. Individual forms of systemic autoimmune disease including CTD, microscopic polyangiitis and Crohn's disease were diagnosed according to the criteria of the corresponding societies. The following were considered autoantibody positive (Ab+) in our study: an antinuclear antibody (ANA) titer greater than 1:320, a positive of anti-Sjogren's syndrome antigen A (SSA) or anti-Sjogren's syndrome antigen B (SSB), anti-Scl-70, anti-Sm, anti-Jo-1, anti-ribonucleoprotein antibody (anti-RNP), anti-keratin antibody (AKA), anti-perinuclear factor (APF), or anti-cyclic citrullinated peptide antibodies (anti-CCP) [27–32]. NSIP patients who meet the criteria for a systemic autoimmune disease (SAD) were defined as the SAD-NSIP group [30, 33–35]; NSIP patients who had at least one positive serologic antibody test were defined as the antibody positive group (i-NSIP-Ab + group); and NSIP patients with negative serologic antibodies tests were defined as the antibody negative group (i-NSIP-Ab- group).

### Clinical characteristics

The patients in this study had the following clinical characteristics documented at the time of their first visit: age, sex, symptoms at the time of the surgical lung biopsy (cough, dyspnea, or wheeze), symptoms or signs of systemic autoimmune disease, smoking status, physical exam findings, arterial blood gas analysis (ABG), and serologic auto-antibody tests.

### Pulmonary function tests

Spirometry, total lung capacity (TLC) by plethysmography, forced vital capacity (FVC) and diffusing capacity of the lung for carbon monoxide (DLCO) were measured according to the ATS recommendations [36–38], and the results were expressed as percentage of the normal predicted values.

### Analysis of subsets of lymphocytes from bronchoalveolar lavage fluid (BALF)

The patients were examined by bronchoscopy with an electric video bronchoscope wedged into the segmental bronchus of the right middle lobe. One hundred milliliters of sterile saline was injected according to the guidelines for the measurement of the cellular components and standardization of BAL [39, 40]. To evaluate the cell subsets, the lymphocytes were stained with anti-CD3, anti-CD4 and anti-CD8 monoclonal antibodies coupled to fluorescein isothiocyanate, and the cellular fluorescence was measured with a FACS Calibur flow cytometer.

### High-resolution CT scanning

High-resolution computed tomography (HRCT) chest scans were performed on all patients at the time of the initial evaluation. The films were reviewed in a blinded fashion by chest radiologists experienced in interpretation of diffuse lung diseases. The specific findings of the HRCT were documented for the index scan (the first scan that documented the presence of ILD). The extent of emphysema, ground-glass opacity (GGO), reticulation, consolidation, and honeycombing (HC) were scored on a scale of 5% for all lobes. HC was defined as clustered cystic airspaces, 3 to 10 mm in diameter, in the subpleural areas of the lungs with well-defined shared walls and layering.

### Treatment and follow-up

After being diagnosed with NSIP, all patients received a course of oral prednisolone, starting at 0.5 mg/kg/d for one month, tapered every 3 weeks to 5-7.5 mg/d and then maintained in 5-7.5 mg/d. The total length of treatment was 12-18 months.

The patients underwent follow-up assessments at the Interstitial Lung Disease Clinic of Peking Union Medical College Hospital 3, 6 and 12 month after surgical lung biopsy, and then once a year. The patients in the SAD-NSIP group were treated combined with cytotoxic drugs.

### Statistical analysis

All values are expressed as the mean ± SD. Student’s t-test was used for analysis of normally distributed data. The Wilcoxon test and Kruskal-Wallis test were used to compare two or more sets of non-normally distributed data, respectively. Fisher's exact test was used to determine group differences. Cox multivariate regression analysis was used to evaluate the survival risk factors. The overall survival durations were calculated by the Kaplan-Meier method. The correlation coefficients were calculated using Spearman’s rank method. The probability values were obtained from 2-sided tests, with a statistical significance of *p* < 0.05. SPSS 15.0 (SPSS for Windows, version 15.0 [SPSS Inc., Chicago, Illinois, USA]) was used for the statistical analyses.

## Results and discussion

Ninety-seven NSIP patients were included. The mean age of the study population was 48 ± 11 years (median 48, range 16 to 69), and 73% were women. The mean follow-up time was 54 ± 34 months (median 45, range 2 to 120).

We divided the NSIP patients into three groups according the above criteria at the time of surgical lung biopsy. The SAD-NSIP group included 23/97(23.7%) of the patients; the i-NSIP-Ab + group included 30/97 (30.9%); and the i-NSIP-Ab- group included 44/97(45.4%). The underlying types of systemic autoimmune disease were rheumatoid arthritis (n = 3), scleroderma (n =3), Sjogren’s syndrome (n =4), polymyositis (n = 9), SLE (n = 1), MPA (n = 2) and ulcerative colitis (n = 1).

At the end of December 2012, typical clinic manifestations of CTD developed in five cases from i-NSIP-Ab + group and i-NSIP-Ab- group. Respiratory symptoms preceded other systemic manifestations by a median of 48 months (range 1-108). Additionally, 4 cases in the i-NSIP Ab- group were later found to have positive autoantibodies during the median follow-up period of 66 months (range 12-108). Three cases were diagnosed as having polymyositis (one case from i-NSIP-Ab + group, two cases from i-NSIP-Ab- group), one as scleroderma (scl-70 positive and skin biopsy) and another one as microscopic polyarteritis (from i-NSIP-AB-group, p-ANCA and MPO positive, renal biopsy). Additionally, three cases in the i-NSIP-Ab- group were later found to have positive autoantibodies. Two cases were ANA positive (one at 1:640; another at 1:1280), and another case was anti-Jo-1 positive.

At the end of December 2012, the underlying types of systemic autoimmune disease were rheumatoid arthritis (n = 3), scleroderma (n =4), Sjogren’s syndrome (n =4), polymyositis (n = 12), SLE (n = 1), MPA (n = 3) and ulcerative colitis (n = 1). We re-classified the NSIP patients according the follow-up results. The post-follow-up distribution of the subjects was: 28/97 patients (28.9%) in the SAD-NSIP group, 31/97 (32.0%) in the i-NSIP-Ab + group and 38/97 (39.1%) in the i-NSIP-Ab- group.

### Comparison of clinical manifestations among the *CTD-NSIP, i-NSIP-Ab + and i-NSIP-Ab- groups*

The demographic characteristics are shown in Table 1. There were no differences among the three groups of the NSIP patients as classified at the time of surgical lung biopsy or after re-classification at the time of follow-up (a median of 45 months).

**Table 1:** **Comparison of demographics among three groups**

	SAD-NSIP group	i-NSIP-Ab + group	i-NSIP-Ab-group	P
	N (%)	N (%)	N (%)	
Patient’s number				
Initial presentation	23(23.7)	30(30.9)	44(45.4)	0.050
After follow up	28(28.9)	31(32.0)	38(39.1)	0.306
Age (years), Mean ± SD				
Initial presentation	49.4 ± 10.6	45.7 ± 10.0	49.5 ± 12.5	0.307
After follow up	50.4 ± 9.9	46.3 ± 10.6	48.5 ± 12.8	0.386
Male				
Initial presentation	6/23(26.1)	6/30(20.0)	15/44(34.1)	0.405
After follow up	8/28(28.6)	7/31(22.6)	13/38(34.2)	0.569
Duration (months), Mean ± SD				
Initial presentation	16.7 ± 27.6	10.2 ± 13.8	10.9 ± 15.4	0.378
After follow up	14.9 ± 25.3	9.8 ± 13.8	11.8 ± 16.3	0.575
Follow-up time (months), Mean ± SD				
Initial presentation	48.4 ± 28.2	58.4 ± 32.7	54.9 ± 38.5	0.504
After follow up	51.9 ± 29.8	58.3 ± 32.5	55.2 ± 38.9	0.745
Smoking,				
Initial presentation	2/23(8.7)	2/30(6.7)	4/44(9.1)	0.944
After follow up	2/28(7.1)	2/31(6.5)	4/38(10.5)	0.803

The demographic characteristics are shown in above Table. There were no differences among the three groups of the NSIP patients as classified at the time of surgical lung biopsy or after re-classification at the time of follow-up.

The clinical characteristics of study subjects are shown in Table 2. Skin rash was strongly associated with the SAD-NSIP and i-NSIP-Ab + groups relative to the i-NSIP-Ab- group as classified at the time of surgical lung biopsy or after re-classification at the time of follow-up.

**Table 2:** **Comparison of clinical features among three groups**

	SAD-NSIP	i-NSIP-Ab+	i-NSIP-Ab-	P
	N (%)	N (%)	N (%)	
Cough, initial presentation	14/23(60.9)	21/30(70.0)	22/44(50.0)	0.150
After follow up	17/28(60.7)	21/31(67.7)	19/38(50.0)	0.215
Dyspnea, initial presentation	13/23(56.5)	21/30(70.0)	34/44(77.3)	0.334
After follow up	17/28(60.7)	22/31(71.0)	29/38(76.3)	0.389
Fever, initial presentation	5/23(21.7)	10/30(33.3)	10/44(22.7)	0.475
After follow up	9/28(36.0)	9/31(29.0)	7/38(18.4)	0.399
Weight loss, initial presentation	1/23(4.3)	7/30(23.3)	8/44(18.2)	0.168
After follow up	3/28(10.7)	8/31(25.8)	5/38(13.2)	0.230
Arthralgia, initial presentation	5/23(21.7)	5/30(16.7)	6/44(13.6)	0.697
After follow up	7/28(25.0)	6/31(19.4)	3/38(7.9)	0.158
Raynaud’s phenomenon, initial presentation	3/23(13.0)	3/30(10)	0	0.063
After follow up	3/28(10.7)	3/31(9.7)	0	0.126
Dry eyes or dry mouth, initial presentation	3/23(13.0)	1/30(3.3)	3(6.8)	0.396
After follow up	3/28(10.7)	1/31(3.2)	3(6.5)	0.529
Oral ulcer, initial presentation	4/23(17.4)	2/30(6.7)	1/44(2.3)	0.075
After follow up	4/28(14.3)	2/31(6.5)	1/38(2.6)	0.191
Skin rash, initial presentation	8/23(34.8)	12/30(40.0)	1/44(2.3)	0.000*
After follow up	8/28(28.6)	13/31(41.9)	0	0.000*
Proximal muscle weakness, initial presentation	3/23(13.0)	0	2/44(4.5)	0.101
After follow up	3/28(10.7)	0	2/38(5.3)	0.178
Morning stiffness, initial presentation	2/23(10.7)	0	0	0.037**
After follow up	2/28(7.1)	0	0	0.081
Gastroesophageal reflux, initial presentation	2/23(8.7)	0	0	0.037**
After follow up	2/28(7.1)	0	0	0.081
Photosensitivity, initial presentation	0	0	1/44(2.3)	0.544
After follow up	0	0	1/38(2.6)	0.456
Crackles, initial presentation	17/23(73.9)	16/30(53.3)	28/44(63.6)	0.173
After follow up	18/28(64.3)	16/31(51.6)	27/38(71.0)	0.343
Clubbing, initial presentation	2/23(8.7)	3/30(10.0)	1/44(2.3)	0.339
After follow up	2/28(7.1)	3/31(9.7)	1/38(2.6)	0.467

*Skin rash was strongly associated with the ASD-NSIP and i-NSIP-Ab + groups relative to the i-NSIP-Ab- group at the time of surgical lung biopsy or after re-classification.

**Morning stiffness and Gastroesophageal reflux were associated with ASD-NSIP at the time of surgical lung biopsy. No differences among the three groups after re-classification at the time of follow-up.

ASD = systemic autoimmune disease.

The laboratory data are shown in Table 3. There were no differences among the three groups either at the time of the surgical lung biopsy or after the post-follow-up reclassification. Pulmonary function, BAL lymphocytes analysis and ABG were similar among the three groups at the time of surgical lung biopsy or after the post-follow-up reclassification.

**Table 3:** **Comparison of LAB findings among three groups**

	SAD-NSIP group	i-NSIP-Ab + group	i-NSIP-Ab-group	p
ABG				
PaO_2_ (mmHg), initial presentation	74.1 ± 8.9	74.2 ± 10.8	74.2 ± 10.8	0.648
After follow up	73.0 ± 10.7	73.2 ± 11.8	73.9 ± 10.6	0.934
PaCO_2_(mmHg), initial presentation	36.8 ± 4.9	35.3 ± 4.2	35.7 ± 4.5	0.478
After follow up	37.1 ± 4.9	35.4 ± 4.2	35.2 ± 4.1	0.206
PFT				
FVC (%), initial presentation	70.1 ± 16.3	72.3 ± 13.5	74.5 ± 12.3	0.789
After follow up	68.9 ± 14.9	74.1 ± 14.7	75.7 ± 14.4	0.664
TLC (%), initial presentation	75.1 ± 12.4	74.1 ± 13.2	73.4 ± 12.1	0.912
After follow up	73.4 ± 11.7	74.4 ± 14.9	76.0 ± 17.6	0.847
DLCO (%), initial presentation	55.1 ± 10.9	53.4 ± 16.2	52.1 ± 15.2	0.198
After follow up	51.4 ± 11.0	54.2 ± 18.0	56.8 ± 16.6	0.173
BALF				
M (%), initial presentation	42.9 ± 21.7	43.8 ± 22.6	45.6 ± 23.7	0.397
After follow up	46.9 ± 24.7	44.0 ± 21.7	43.6 ± 24.3	0.425
L (%), initial presentation	39.2 ± 20.9	38.9 ± 22.4	41.3 ± 23.5	0.876
After follow up	37.1 ± 22.8	38.4 ± 20.7	44.8 ± 25.0	0.776
N (%), initial presentation	10.1 ± 18.2	13.6 ± 14.2	12.1 ± 11.9	0.671
After follow up	11.9 ± 19.6	13.2 ± 15.7	10.5 ± 13.1	0.807
E (%), initial presentation	5.0 ± 8.8	5.2 ± 6.0	4.1 ± 5.0	0.801
After follow up	5.3 ± 8.2	4.8 ± 5.7	3.9 ± 4.6	0.752
CD4/CD8, initial presentation	1.5 ± 1.9	1.3 ± 1.3	1.2 ± 0.9	0.813
After follow up	1.2 ± 1.8	1.2 ± 1.1	1.4 ± 1.5	0.947

ABG, PFTs and BAL lymphocytes analysis were similar among the three groups. There were no differences among the three groups either at the time of the surgical lung biopsy or after the post-follow-up reclassification.

ABG = arterial blood gas analysis; BAL = Bronchial alveolus lavage; PFT = pulmonary function tests.

#### Radiologic and pathologic findings in CTD-NSIP, i-NSIP-Ab + and i-NSIP-Ab- groups

The characteristic features of the HRCT for the three groups are shown in Table 4. No statistically significant differences were observed among the three groups of patients as classified at the time of surgical lung biopsy or after the post-follow-up reclassification.

**Table 4:** **Comparison of chest CT and pathological findings among three groups**

	SAD-NSIP	i-NSIP-Ab+	i-NSIP-Ab-	P
	n (%)	n (%)	n (%)	
**Baseline Chest CT Findings**				
Ground glass opacity, initial presentation	17/23(73.9)	20/30(66.7)	25/44(56.8)	0.358
After follow up	19/28(67.9)	21/31(67.7)	23/38(60.5)	0.765
Patchy opacity, initial presentation	9/23(39.1)	10/30(33.3)	12/44(27.2)	0.602
After follow up	10/28(35.7)	11/31(35.5)	10/38(26.3)	0.633
Reticular opacity, initial presentation	18/23(78.2)	19/30(63.3)	27/44(61.4)	0.358
After follow up	22/28(78.6)	20/31(64.5)	22/38(57.9)	0.211
Traction bronchiectasis, initial presentation	9/23(39.1)	6/30(20.0)	8/44(18.2)	0.167
After follow up	10/28(35.7)	6/31(19.4)	7/38(18.4)	0.208
**Pathological pattern**				
Cellular pattern, initial presentation	12/23(52.2)	15/30(50.0)	22/44(50.0)	0.984
After follow up	15/28(53.6)	14/31(45.2)	20/38(52.6)	0.768
Mixed pattern, initial presentation	8/23(34.8)	13/30(43.3)	17/44(38.6)	0.815
After follow up	10/28(35.7)	14/31(45.2)	14/38(36.8)	0.707
Fibrotic pattern, initial presentation	3/23(13.0)	2/30(6.7)	5/44(11.4)	0.716
After follow up	3/28(10.7)	4/31(12.9)	3/38(7.9)	0.791

The characteristic features of HRCT and histo-pathological pattern of the three groups are seen in this table. No statistically significant differences were observed among the three groups as classified at the time of surgical lung biopsy or after the post-follow-up reclassification.

The histological analysis is shown in Table 4. Additionally, no statistically significant differences were observed among the three subgroups of patients as classified at the time of surgical lung biopsy or after the post-follow-up reclassification.

#### Treatment and survival

**Figure 1:**
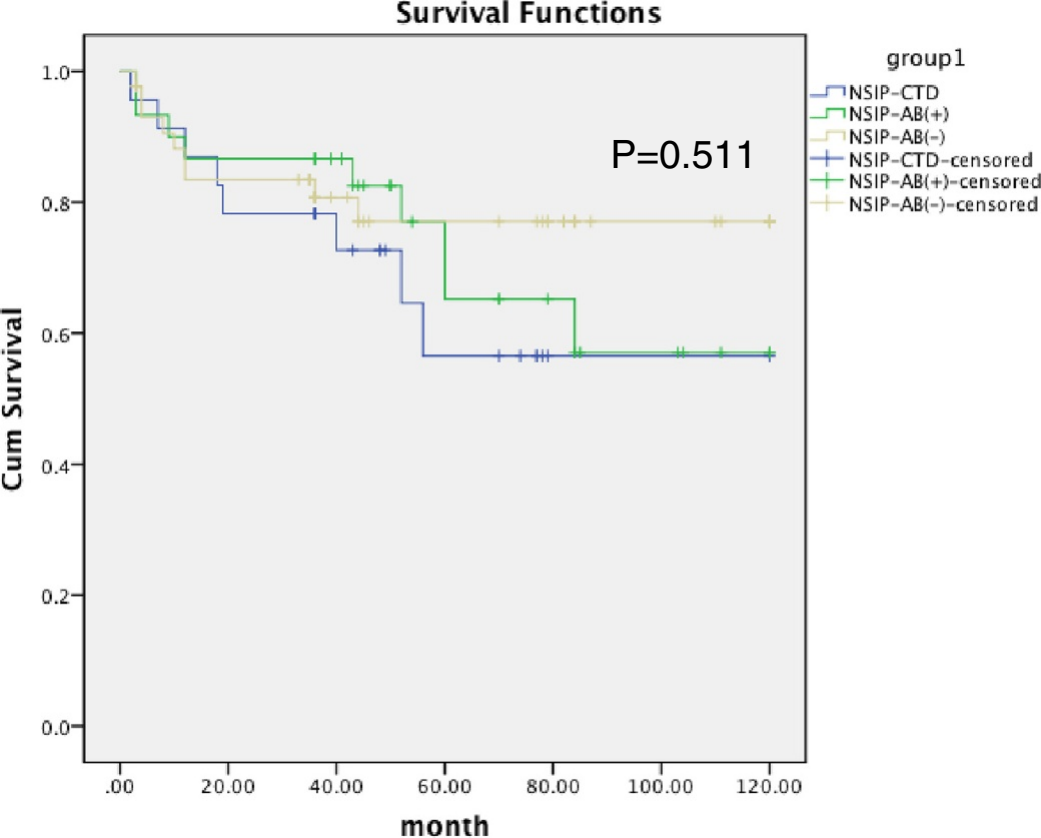
**Kaplan-Meier survival curve for NSIP patients divided by clinical status at intital: SAD-NSIP, i- NSIP-AB + and i-NSIP-Ab- patients at the time of surgical lung biopsy.** There is no significant difference (p = 0.511) among three groups (Log Rank = 1.342).

**Figure 2:**
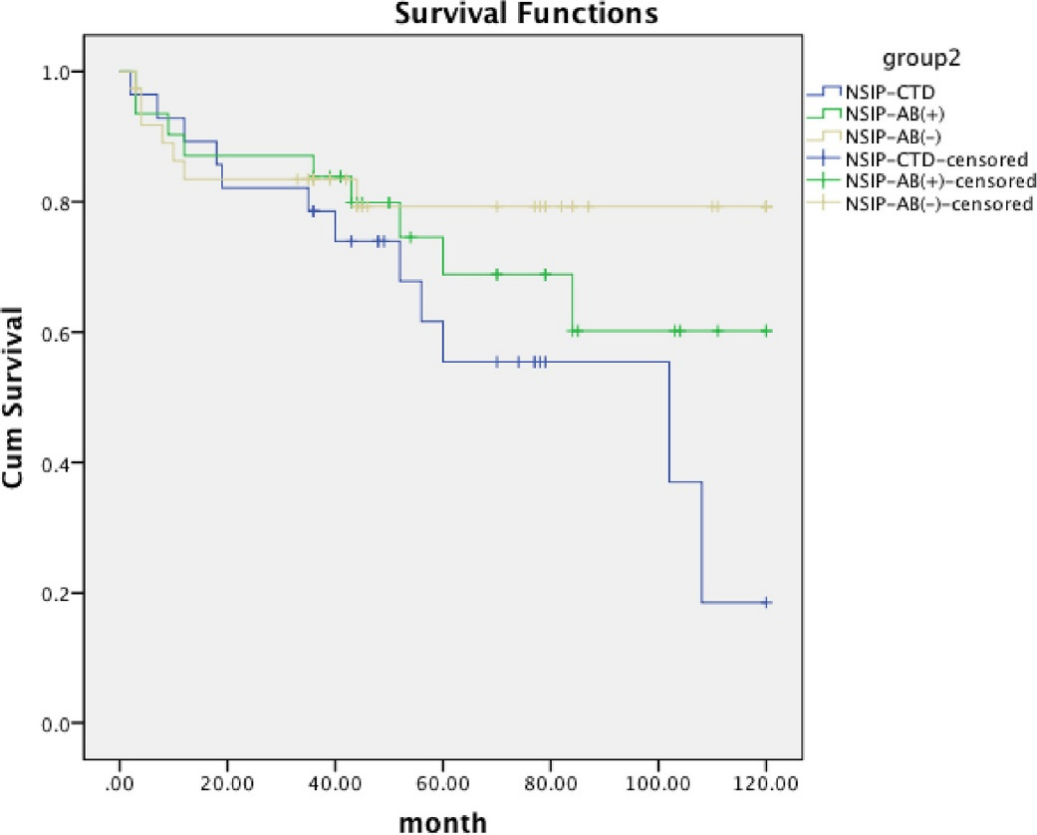
**Kaplan-Meier survival curve for NSIP patients re-divided by clinical status after follow up 54 ± 34 months.** ASD-NSIP *vs* i-NSIP-Ab- =0.059; ASD-NSIP *vs* i-NSIP-Ab + =0.232; i-NSIP-Ab + *vs* i-NSIP-Ab- =0.456.

**Figure 3:**
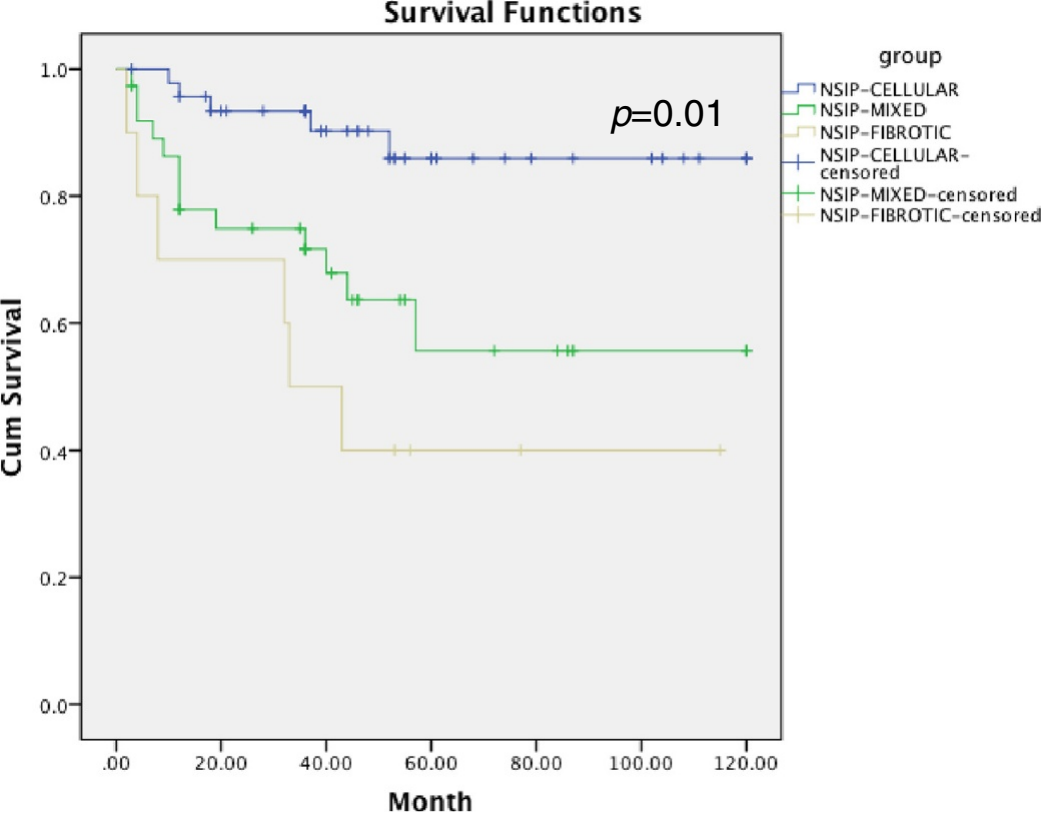
**Kaplan-Meier survival curve for NSIP patients divided by pathological pattern: cellular pattern** ***vs*** **mixed pattern =0.001; cellular pattern** ***vs*** **fibrotic pattern =0.000; Mixed pattern** ***vs*** **fibrotic pattern =0.451.**

The treatment and follow-up results are summarized in Table 5.Based on the classification at the time of surgical lung biopsy, there were no significant differences among the three groups, p = 0.511 (Kaplan-Meier survival curves are shown in Figure 1). The diagnosis of systemic autoimmune disease was not associated with poorer survival (HR, 0.368, 95% CI, 0.680-3.067; p = 0.339). However, based on the classification after the follow-up period, a marginally significant statistical difference could be observed between the ASD-NSIP and NSIP-Ab- groups (Figure 2, p = 0.059). Systemic autoimmune disease was an independent risk factor for the survival of patients with NSIP after follow-up (HR, 0.471; 95% CI, 0.246-0.901; p = 0.023).Pathological pattern was associated with the survival time, p = 0.010 (Kaplan-Meier survival curves are shown in Figure 3). There is a significant difference (p = 0.01) among three groups (Log Rank=13.391). Fibrotic pattern was an independent risk factor for the survival of patients with NSIP (HR, 0.316; 95% CI, 0.115-0.870; p = 0.026).

**Table 5:** **Comparison of treatment and outcomes among three groups**

	SAD-NSIP group	i-NSIP-Ab + group	i-NSIP-Ab-group
Initial therapy			
Prednisone, N (%)	9/23(39.1)	30/30(100)	44/44(100)
Prednisone + immunosuppressive agent, N (%)	14/23(60.9)	0	0
Relapse, N (%)	6/23(26.1)	13/30(4.3)	21/44(47.7)
Emerging autoantibody, N (%)	0	0	3/44(6.8)
classifiable SAD, N (%)	0	2/30(6.7)	3/44(6.8)
PAH, N (%)	1/23(4.3)	1/30(3.3)	1/44(2.3)
Lung infection, N (%)	1/23(4.3)	3/30(10.0)	4/44(9.1)
Death, N (%)	8/23(34.8)	8/30(26.7)	9/44(20.5)
Underlying disease, N (%)	4/23(17.4)	6/30(20.0)	6/44(13.6)
Lung infection, N (%)	3/23(13.0)	2/30(6.7)	2/44(4.5)
Other, N (%)	1/23(4.3)	0	1/44(2.3)

Smoking (HR, 0.964; 95% CI, 0.348-2.679; p = 0.943), TLC (HR, 1.013; 95% CI, 0.991-1.036; p = 0.260), FVC (HR, 1.032; 95% CI, 0.997-1.067; p = 0.764) and DLCO (HR, 0.978; 95% CI, 0.945-1.012; p = 0.206) were not the risk factor for survival.

Our study revealed a total of 5/74 (6.8%) cases were diagnosed as having systemic autoimmune disease after follow-up. Romagnoli’s study showed that 3 of 27 (11%) i-NSIP patients were diagnosed as having CTD after a follow-up of 59.7 months [13]. Park reported that 8/87 cases (10%) of i-NSIP patients developed CTD during a median follow-up of 53 months [14]. The prevalence in these previous studies was higher than in our study. One reason for the difference could be that the median follow-up time in our study was 45 months, but the typical systemic autoimmune disease symptoms, signs and serum biomarkers were generally first observed at times between 48 and 66 months of follow-up. It is therefore possible that some of the i-NSIP patients might have developed typical systemic autoimmune disease after our follow-up period.

We reviewed the published papers that compared survival between CTD-ILD and IIP and found that all of the studies classified the patients according to the patient’s presentations at the time of the first visit. The results indicated that the clinical and radiologic features of CTD-NSIP were similar to idiopathic NSIP [22, 23], and CTD did not affect survival in NSIP patients [24, 26]. In our study, we followed the methods of the previous studies and divided the patients according the clinical manifestation at the time of surgical lung biopsy. The results were consistent with the previous studies in showing no differences in survival time among those three NSIP classifications [18, 41, 42]. i-NSIP-Ab + was also not associated with a survival benefit according to several studies [18, 23, 43]. Then, we re-classified the NSIP patents according the follow-up results and compared the patients’ clinical manifestations, radiographic findings and pathological features. There were still no differences among three groups after follow-up. The patients who were defined as having systemic autoimmune disease associated NSIP could not be distinguished from those who were defined as i-NSIP with or without antibodies based on pulmonary manifestations or respiratory physiology even after follow-up. This result indicates that recognition of systemic autoimmune disease is particularly challenging in NSIP [25, 26, 44, 45].

Using Cox multivariate analysis, we found that systemic autoimmune disease was a risk factor for survival. Furthermore, we found that the survival times for the SAD-NSIP patients were shorter than for those classified as i-NSIP because some i-NSIP patients with poor prognoses were eventually diagnosed as having systemic autoimmune disease and were re-assigned to the SAD-NSIP group. Lee’s study showed that the patients with NSIP and various systemic conditions had worse prognoses. However, no statistically significant relationship was found between the systemic conditions and poor prognosis in that study [42]. Felício [24] detected significantly greater collagen and elastic fiber production in the lungs of patients with CTD-NSIP compared with those with idiopathic NSIP [24]. The increased elastin content may have been caused by major repair and remodeling processes following septal inflammation and consequent fiber fragmentation in CTD-NSIP. These processes might be responsible for the worse prognosis. However, the specific mechanism is currently uncertain. More prospective studies with larger numbers of subjects are required.

Our study was limited by its retrospective nature, and because this was a retrospective study, the subjects were restricted to the patients who had undergone a surgical lung biopsy. In addition, the follow-up period might not have been long enough to clearly show differences in the prognosis between SAD-NSIP and i-NSIP. Although the number of subjects in our study was relatively large, it might not have been enough to clearly show the differences between SAD-NSIP and i-NSIP. Further studies of larger numbers of subjects, perhaps through multicenter cooperation, will be required to overcome this limitation. Nevertheless, this is the first time that the NSIP patients were re-classified after the follow-up period. We believe that the data presented here remain valid despite these shortcomings.

## Conclusion

Based on the results of our study, we concluded that some idiopathic NSIP cases may represent the first manifestation of an underlying systemic autoimmune disease. Long-term follow-up of patients with idiopathic NSIP is recommended.

## Abbreviations

AKA: Anti-keratin antibody
ANA: Anti-nuclear antibody
APF: Anti-perinuclear factor
ATS: American Thoracic Society
BALF: Bronchoalveolar lavage Fluid
CCP: Anti-cyclic citrullinated peptide antibodies
CTD: Connective tissue disease
CBC: Complete blood count
DLCO: Diffusing capacity of the lung for carbon monoxide
ERS: European Respiratory Society
ESR: Erythrocyte sedimentation rate
FVC: Forced vital capacity
HRCT: High-resolution computed tomography
ILD: Interstitial lung diseases
IIP: Idiopathic interstitial pneumonitis
NSIP: Nonspecific interstitial pneumonitis
RA: Rheumatoid arthritis
RNP: Anti-ribonucleoprotein antibody
SAD: Systemic autoimmune disease
SSA: Anti-Sjogren's syndrome antigen A
SSB: Anti-Sjogren's syndrome antigen B
UIP: Usual interstitial pneumonia.
